# Melatonin reverses nasopharyngeal carcinoma cisplatin chemoresistance by inhibiting the Wnt/β-catenin signaling pathway

**DOI:** 10.18632/aging.102968

**Published:** 2020-03-23

**Authors:** Jian Zhang, Tao Xie, Xi Zhong, Hua-Li Jiang, Rong Li, Bai-Yao Wang, Xiao-Ting Huang, Bo-Hong Cen, Ya-Wei Yuan

**Affiliations:** 1 Department of Radiation Oncology, Affiliated Cancer Hospital and Institute of Guangzhou Medical University, State Key Laboratory of Respiratory Diseases, Guangzhou Institute of Respiratory Disease, Affiliated Cancer Hospital and Institute of Guangzhou Medical University, Guangzhou, P. R. China; 2 Department of Radiology, Affiliated Cancer Hospital and Institute of Guangzhou Medical University, Guangzhou, P. R. China; 3 Department of Cardiovascularology, Tungwah Hospital of Sun Yat-Sen University, Dongguan, P.R. China

**Keywords:** chemoresistance, cisplatin, melatonin, nasopharyngeal carcinoma, Wnt/β-catenin

## Abstract

Cisplatin (DDP)-based concurrent chemo-radiotherapy is a standard approach to treat locoregionally advanced nasopharyngeal carcinoma (NPC). However, many patients eventually develop recurrence and/or distant metastasis due to chemoresistance. In this study, we aimed to elucidate the effects of melatonin on DDP chemoresistance in NPC cell lines in *vitro* and *vivo*, and we explored potential chemoresistance mechanisms. We found that DDP chemoresistance in NPC cells is mediated through the Wnt/β-catenin signaling pathway. Melatonin not only reversed DDP chemoresistance, but also enhanced DDP antitumor activity by suppressing the nuclear translocation of β-catenin, and reducing expression of Wnt/β-catenin response genes in NPC cells. In *vivo*, combined treatment with DDP and melatonin reduced tumor burden to a greater extent than single drug-treatments in an orthotopic xenograft mouse model. Our findings provide novel evidence that melatonin inhibits the Wnt/β-catenin pathway in NPC, and suggest that melatonin could be applied in combination with DDP to treat NPC.

## INTRODUCTION

Nasopharyngeal carcinoma (NPC) is a highly invasive and metastatic head and neck cancer that is prevalent in Southeast Asia, especially southern China. Due to the lack of early symptoms and the aggressiveness of NPC [1], more than 70% of NPC patients present with locoregionally advanced stage at the initial visit, and often have poor disease prognosis [2, 3]. It is essential to elucidate the molecular mechanisms that regulate NPC progression in order to develop novel treatment strategies.

Cisplatin (DDP) is a DNA damaging chemotherapeutic agent, and exerts cytotoxicity and/or induction of apoptosis by forming DNA adducts or by targeting therapeutically relevant cancer signaling pathways [4–6]. DDP is a highly effective treatment for various cancers, including NPC. Platinum-based concurrent chemo-radiotherapy is the standard of care for patients with locoregionally advanced nasopharyngeal carcinoma (LA-NPC) [7, 8]. However, some patients with NPC do not benefit from treatment, and suffer adverse side effects of the additional chemotherapy [9–11], and many patients acquire DDP resistance during chemotherapy for NPC.

Melatonin is a pleiotropically-acting molecule that possesses anti-inflammatory, anti-proliferative, and antioxidant activities in *vitro* and *vivo* [12–16]. Melatonin shows potential as a therapeutic agent, because it not only has synergistic effects with chemotherapeutic drugs, but it can also reduce the side effects of anticancer drugs by neutralizing singlet oxygen species, superoxide anion radicals, peroxyl radicals, and hydrogen peroxide [17–20]. However, the precise therapeutic roles and underlying mechanisms of melatonin activity in NPC are unclear, and it is unknown whether melatonin could attenuate DDP resistance.

In the present study, we explored aberrant signaling pathways mediating DDP chemoresistance in NPC cells, we evaluated whether melatonin may possess anticancer activity on NPC cells, and we investigated whether combinatorial treatment of DDP and melatonin is effective in an orthotopic xenograft mouse model of NPC. We found that melatonin inhibited tumorigenesis of NPC cells and reversed DDP resistance both in *vitro* and *vivo* by inhibiting the Wnt/β-catenin signaling pathway. These results suggest that melatonin may be a useful agent to overcome chemoresistance in the treatment of NPC.

## RESULTS

### Activation of the Wnt/β-catenin pathway mediates DDP chemoresistance in NPC

**Figure 1:**
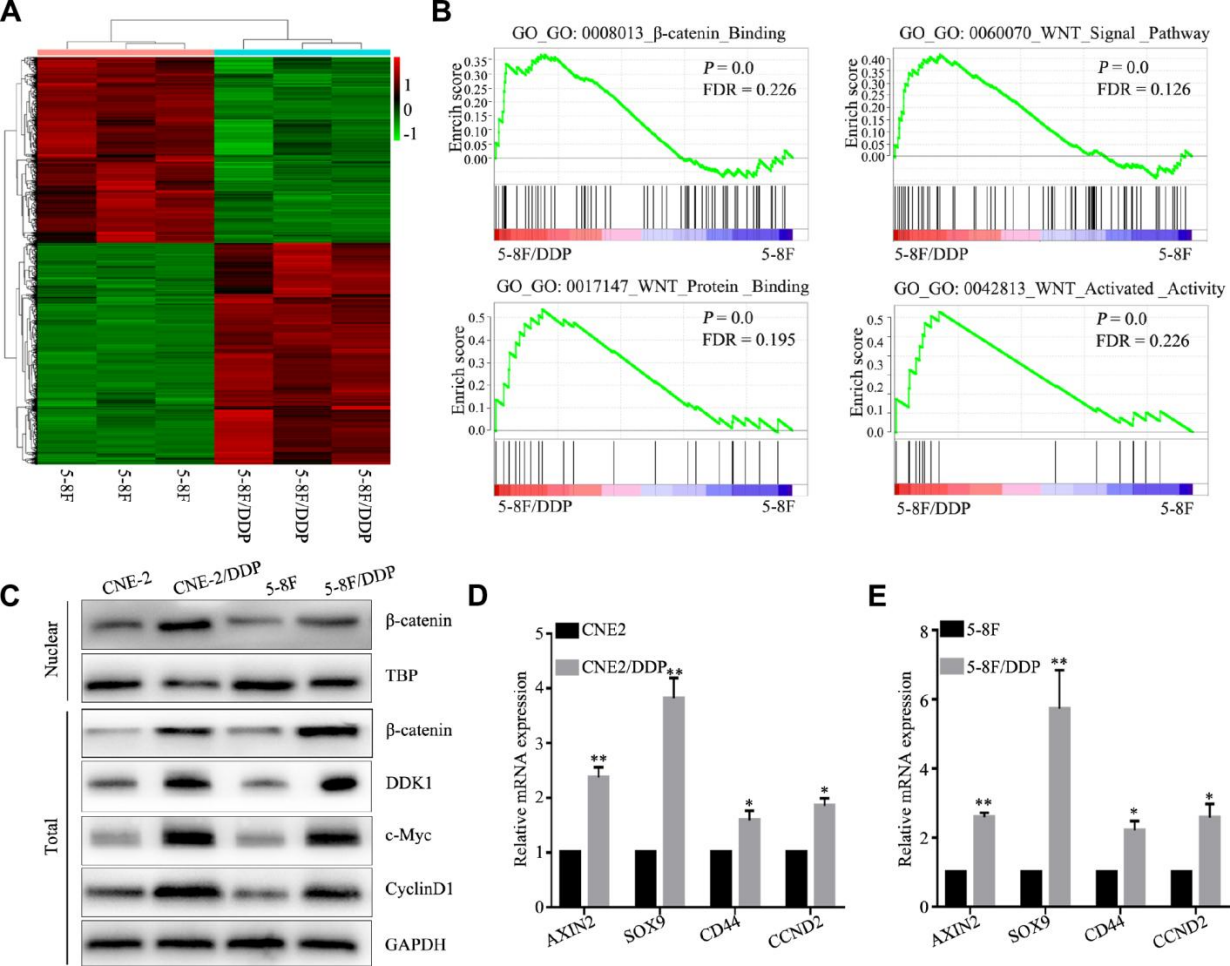
**DDP chemoresistance in NPC cells is mediated through activation of the Wnt/β-catenin pathway.** (**A**) Heatmap of differentially expressed genes in 5-8F and 5-8F/DDP cell lines by RNA sequencing. (**B**) Differentially enriched Wnt/β-catenin pathway-related signatures between 5-8F and 5-8F/DDP cell lines, determined by gene set enrichment analysis (GSEA). (**C**) Protein expression of Wnt/β-catenin in the CNE2 and CNE2/DDP, 5-8F and 5-8F/DDP cell lines, as determined by western blot. (**D**, **E**) mRNA expression of the Wnt/β-catenin downstream genes (AXIN2, SOX9, CD44 and CCND2) in CNE2 and CNE2/DDP cell lines (**D**) and 5-8F and 5-8F/DDP cell lines (**E**) as determined by qPCR. All of the experiments were performed at least three times. Data presented are the mean ± SD; **P < 0.01 compared with using the Student *t*-test.

To know genomic changes in DDP-resistant cells, we established the DDP-resistant cells, 5-8F and CNE2 cell lines were exposed to increasing concentrations of DDP for more than 6 months. Compared with their parental cells, chemoresistance to DDP was observed in 5-8F/DDP and CNE2/DDP cells. As shown in Supplementary Figure 1, 2.5 μg/mL DDP led to almost 50% cell growth inhibition in both 5-8F and CNE2 cells, respectively. Thus, the resistant NPC cells were continuously maintained in RPMI 1600 medium containing 2.5 μg/mL DDP. DDP-resistant were collected to perform a standard RNA sequencing analysis. RNA sequencing analysis identified 3230 mRNAs differentially expressed in DDP-resistant cells compared to DDP-sensitive cells; 1,757 genes were upregulated and 1,473 genes were downregulated (GSE135083) (Figure 1A).

To identify signaling pathways involved in mediating DDP chemoresistance, we performed bioinformatic pathway analysis of differentially expressed genes between DDP-resistant and DDP-sensitive NPC cells. GO interaction analysis identified Wnt signaling related genes, including DKK1, Wnt7B, Wnt10A, FZD1, FZD2, FZD4, FZD7, FZD8 which likely play important roles in regulating sensitivity to DDP chemotherapy (Supplementary Figure 2). To further explore the mechanisms underlying DDP chemoresistance in NPC, we evaluated molecular pathways associated with DDP chemoresistance. GSEA identified gene sets related to activation of the Wnt/β-catenin signaling pathway, including β-catenin binding (GO: 0008013), Wnt signal pathway (GO: 0060070), Wnt protein binding (GO: 0017147) and Wnt activated activity (GO: 0042813) (Figure 1B; *P* < 0.05). These results indicate that DDP chemoresistance in NPC cells is associated with the Wnt/β-catenin signaling pathway.

Western blot analysis validated differential activation of the Wnt/β-catenin signaling pathway in DDP-resistant cells, and showed an increase in nuclear β-catenin in CNE2 DDP chemoresistant cells (CNE2/DDP) and 5-8F DDP chemoresistant cells (5-8F/DDP) (Figure 1C). Similarly, genes downstream of the Wnt/β-catenin signaling pathway were significantly upregulated in CNE2/DDP and 5-8F/DDP cells, including AXIN2, SOX9, CD44, and CCND2 (Figure 1D, 1E). These data illustrate that the activation of Wnt/β-catenin mediates cisplatin chemoresistance in NPC.

### Melatonin suppresses the nuclear translocation of β-catenin in DDP chemoresistant cells

**Figure 2:**
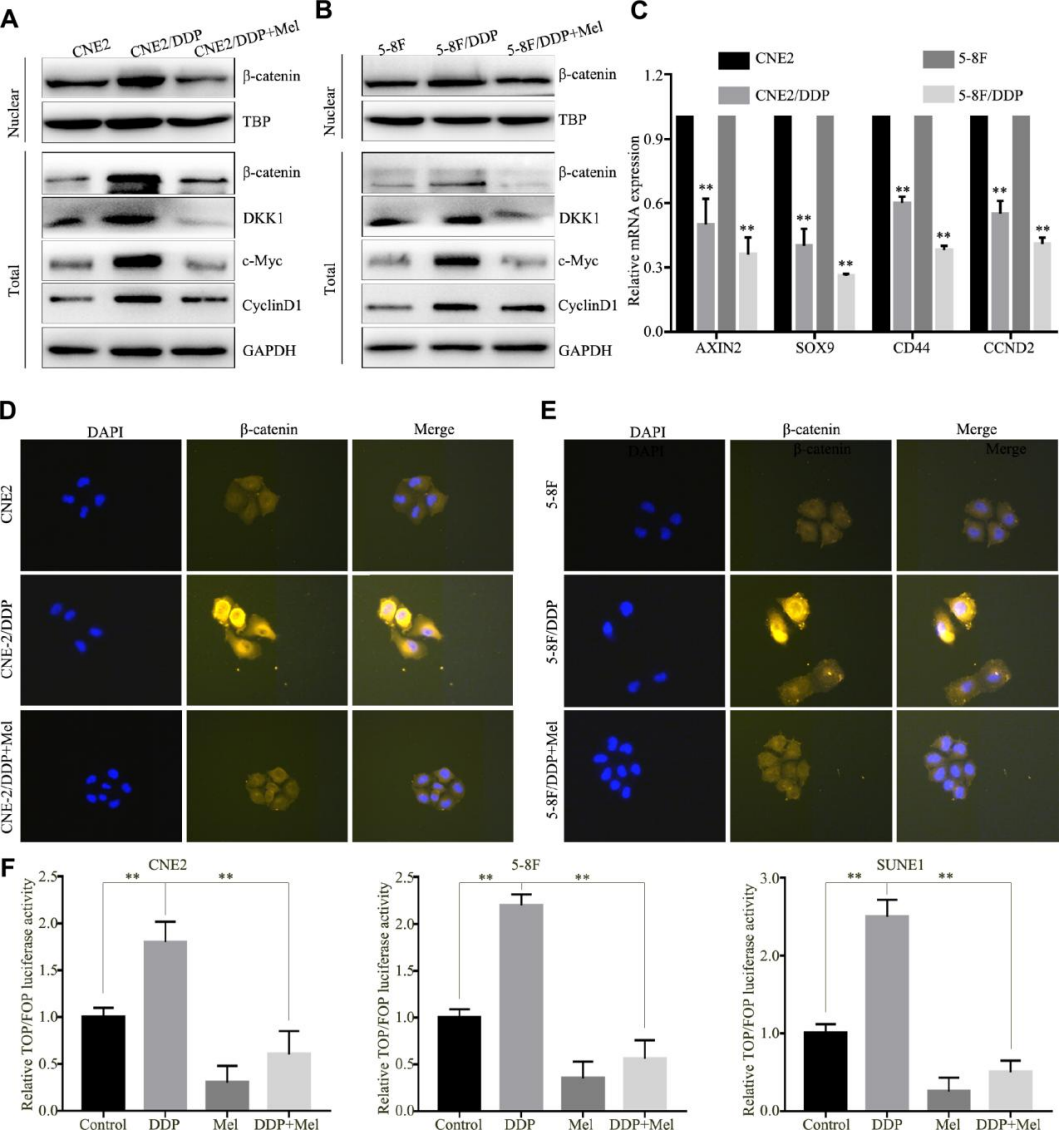
**Melatonin reverses DDP chemoresistance by inhibiting β-catenin nuclear translocation.** (**A**, **B**) Protein expression of β-catenin, DKK1, c-Myc and CylincD1 in the CNE2 and CNE2/DDP (**A**), 5-8F and 5-8F/DDP cell lines (**B**) treated with melatonin (2 mM) for 48 hr, as determined by western blot. (**C**) mRNA expression of Wnt/β-catenin downstream genes (AXIN2, SOX9, CD44 and CCND2) in CNE2 and CNE2/DDP, 5-8F and 5-8F/DDP cell lines treated with melatonin (2 mM) for 48 hr, as determined by qPCR. (**D**, **E**) Representative images of immunofluorescent staining for β-catenin in CNE2 and CNE2/DDP (**D**), 5-8F and 5-8F/DDP (**E**) treated with or without melatonin (2 mM) for 48 hr. (**F**) Relative luciferase activity of NPC cells transfected with the TOP/FOPFlash vector and pRL-TK vector. Data presented are the mean ± SD; **P < 0.01 compared with control using Student *t*-test.

To determine whether melatonin reversed the DDP chemoresistance, CNE2/DDP and 5-8F/DDP cells were treated with 2 mM melatonin for 48 hr. Western blot analysis showed that melatonin decreased the expression of nuclear β-catenin (Figure 2A, 2B). β-catenin functions as a key nuclear effector that controls canonical Wnt signaling in tumorigenesis and metastasis [21–23]. Upon activation of the Wnt pathway, β-catenin translocates into the nucleus and forms β-catenin/TCF complexes, leading to activation of Wnt-mediated gene expression. As shown in Figure 2A and 2B, the downstream genes, c-Myc and CyclinD1, were significantly downregulated in DDP-resistant cells after melatonin treatment. To further verify these findings, we performed qPCR to evaluate the mRNA expression of Wnt pathway genes. Melatonin treatment resulted in downregulation of AXIN2, SOX9, CD44, and CCND2 (Figure 2C), indicating that melatonin may reverse DDP chemoresistance by inhibiting the Wnt/β-catenin pathway.

Immunofluorescence staining showed that DDP-induced accumulation of nuclear β-catenin was decreased in CNE2/DDP and 5-8F/DDP cells following treatment with 2 mM melatonin (Figure 2D, 2E). Moreover, To determine the effects of the melatonin and DDP on on the Wnt signaling pathway, the ratio of the activity of a β-catenin-responsive promoter containing TCF binding sites (TOP-flash) to the activity of a promoter containing mutated TCF binding sites (FOP-flash) was determined in the presence of melatonin or DDP. The expression of β-catenin increased the ratio of TOP-flash activity to FOP-flash activity was considered 100% activity in control group. DDP significantly increased β-catenin activation of TCF-mediated transcription. Melatonin not only decreased β-catenin activation of the TCF promoter, but also reversed the DDP mediated the activation of β-catenin (Figure 2F). Altogether, these data suggest that melatonin reverses the DDP chemoresistance by inhibiting the nuclear translocation of β-catenin.

### Melatonin enhances apoptosis and suppresses metastatic behavior in NPC cells

**Figure 3:**
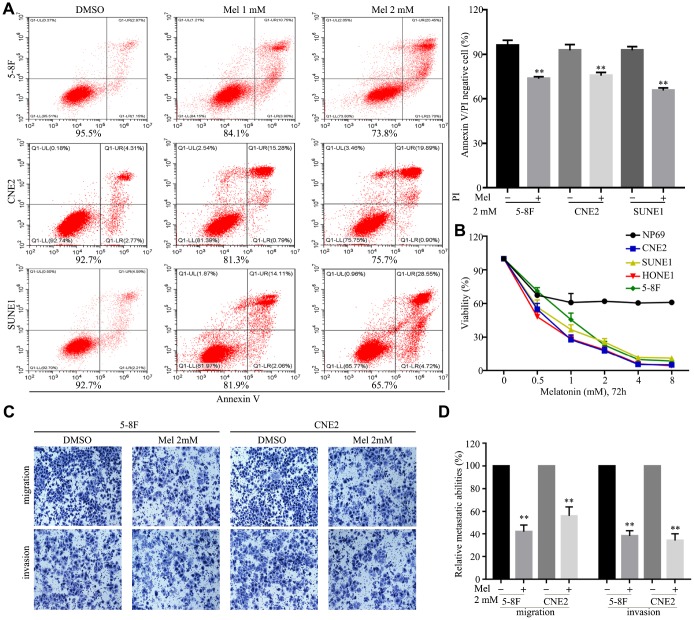
**Melatonin inhibits malignant properties of NPC cells.** (**A**) Representative images (left panel) and quantification (right upper panel) of cell apoptosis in the indicated cells treated with melatonin (Mel, 48 hr), as determined by AnnexinV/propidium iodide (PI) assay. (**B**) The cell viability of the indicated cells incubated with melatonin (72 hr) was determined by CCK-8 assay. (**C**, **D**) Images and quantification of migrated (**C**) and invaded (**D**) NPC cells treated with melatonin (2 mM) for 24 hr were analyzed in transwell assays. Data presented are the mean ± SD; **P < 0.01 compared with control using Student *t*-test.

Melatonin effectively induced cell death and suppressed cell growth in 5-8F, CNE2 and SUNE1 cells in a dose-dependent manner (Figure 3A, 3B), while the NP69 nasopharyngeal epithelial cell line was less sensitive to melatonin. Transwell assays demonstrated that treatment with 2 mM melatonin suppressed migration and invasion of 5-8F and CNE2 cells (Figure 3C, 3D). These results show melatonin can inhibit malignant characteristics of NPC cells.

### Melatonin reverses DDP chemoresistance in NPC DDP-resistant cells

**Figure 4:**
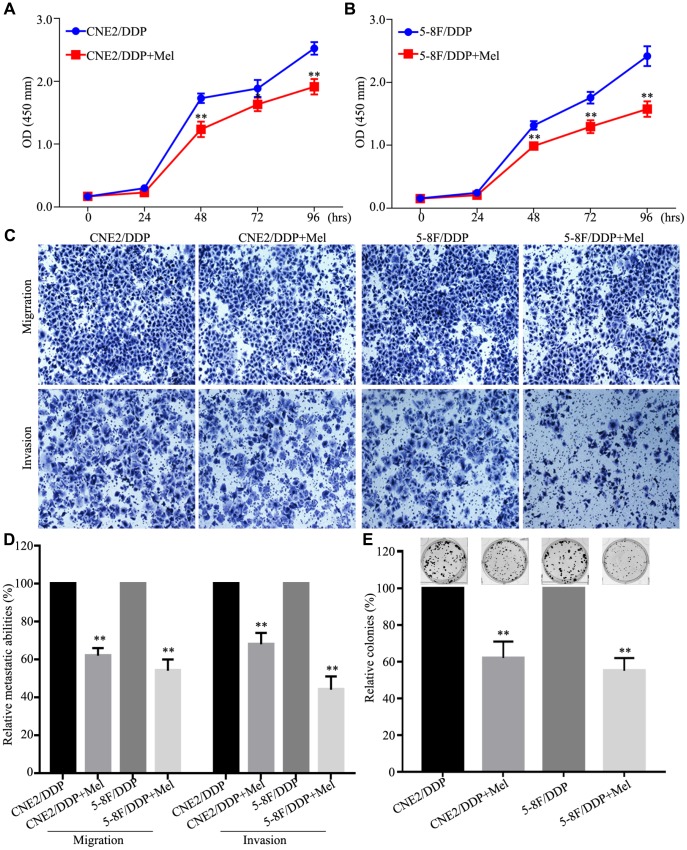
**Melatonin reverses DDP chemoresistance in NPC DDP-resistant cells.** (**A**, **B**) The cell viability of the CNE2/DDP (**A**) and 5-8F/DDP (**B**) cells treated with melatonin (2 mM) was determined by CCK-8 assay. (**C**, **D**) Images (**C**) and quantification (**D**) of transwell migration and invasion assays. (**E**) Images (up) and quantification (down) clonogenic colony formation assays of the CNE2/DDP and 5-8F/DDP cells treated with melatonin for 2 weeks. All of the experiments were performed at least three times. Data presented are the mean ± SD; **P < 0.01, *P < 0.05 compared with control using Student *t*-test.

To further know the function of melatonin on DDP-resistant cells, CNE2/DDP and 5-8F/DDP cells were further treated with 2 mM melatonin. As shown in Figure 4A and 4B, Melatonin significantly inhibited cell growth in CNE2/DDP and 5-8F/DDP cells after treated with melatonin 48h, 72h and 96h. Transwell assays showed that the migration and invasion abilities were significantly decreased after melatonin treatment (Figure 4C, 4D). Colony formation assay further indicated that melatonin could significantly reduce the colony growth in CNE2/DDP and 5-8F/DDP cells (Figure 4E). These results indicate that melatonin can reverses DDP chemoresistance in NPC cells.

### Melatonin and DDP combination inhibits NPC cell growth

**Figure 5:**
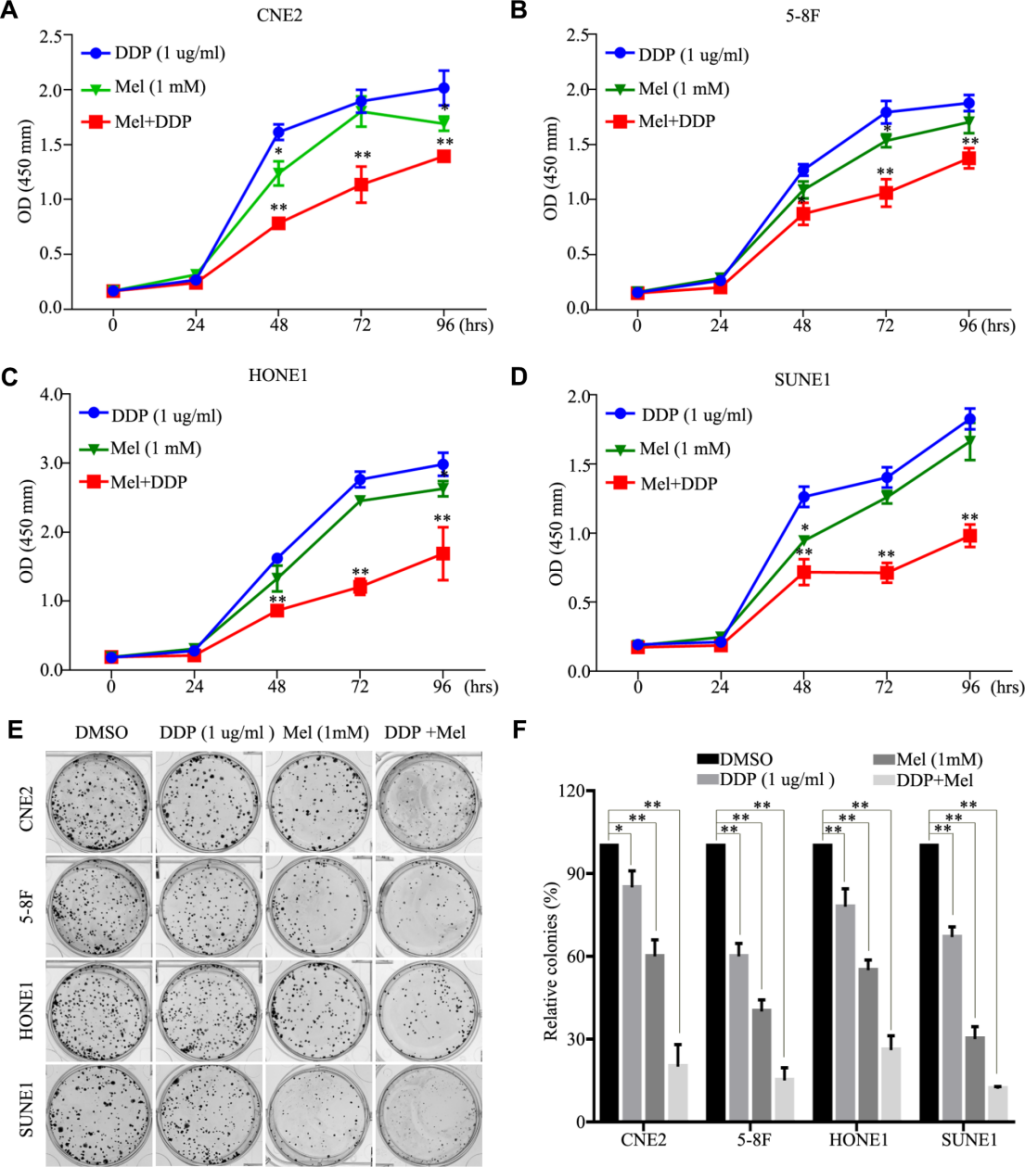
**Effects of melatonin and DDP combination on malignant properties of nasopharyngeal carcinoma (NPC) cells.** (**A**–**D**) The cell viability of the CNE2 (**A**), 5-8F (**B**), HONE1 (**C**) and SUNE1 (**D**) cells incubated with melatonin (72 hr) (green line) alone and DDP alone (blue line) or melatonin with DDP combination (red line) was determined by CCK-8 assay. (**E**, **F**) Images (**E**) and quantification (**F**) clonogenic colony formation assays of the indicated cells treated with melatonin for 2 weeks. All of the experiments were performed at least three times. Data presented are the mean ± SD; **P < 0.01, *P < 0.05 compared with control using Student *t*-test.

The effects of melatonin treatment on cell growth and DDP sensitivity were evaluated in four NPC cell lines: CNE2, 5-8F, HONE1, and SUNE1. After four days of culture with 1 μg/ml DDP, the growth of all NPC cell lines was significantly suppressed. Additionally, the growth was further suppressed after addition of 1 mM melatonin treatment (Figure 5A–5D), suggesting that melatonin and DDP may synergize to suppress growth of NPC cells. Treatment with either melatonin or DDP alone delayed colony growth in CNE2, 5-8F, HONE1, and SUNE1 cells, and treatment with a combination of DDP and melatonin showed synergistic suppression of colony formation (Figure 5E, 5F). These results suggest that melatonin enhances the inhibition of cell proliferation induced by DDP in NPC cell lines.

### Melatonin and DDP combination inhibits NPC tumor metastasis *in vivo*

**Figure 6:**
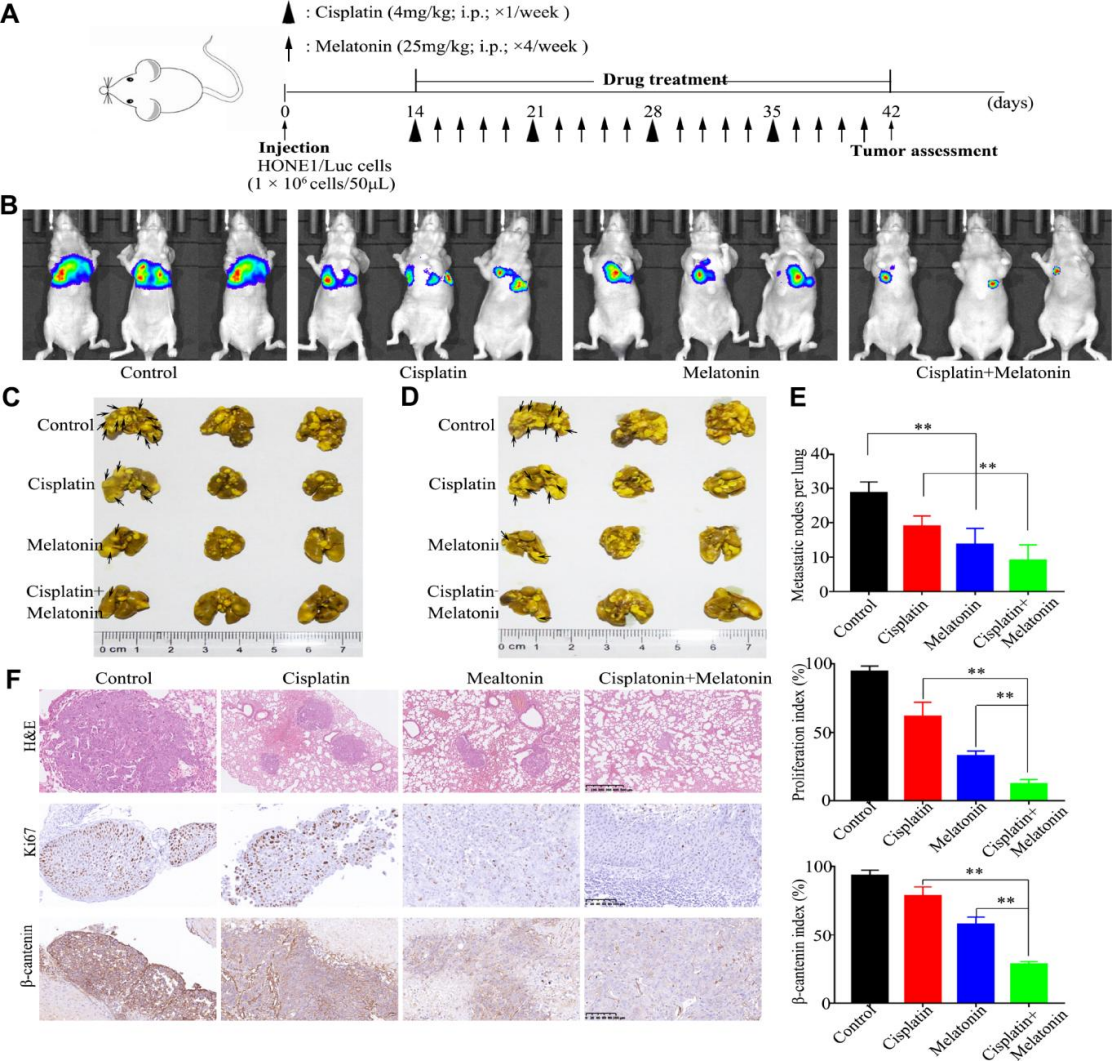
**Antitumor activity of melatonin plus DDP *in vivo* in a lung metastasis model.** (**A**) Tumor progression timeline with experimental treatment time points. (**B**) Tumor cell luminescence from each mouse in each group was acquired on treatment day 28. (**C**, **D**) Representative front images (**C**) and back images (**D**) of macroscopic lung metastases, arrowheads indicate the metastatic nodes. (**E**) Quantification of the average number of macroscopic metastatic nodes formed on the lung surface. (**F**) Paraffin-embedded tumor sections were stained with H&E, anti-Ki67, and β-catenin antibodies (Scar bar: 100 μm). Right panel: quantification of the proliferation index and β-catenin index in tumor sections. These animal experiments were repeated once (n = 3 mice per treatment group). Data presented are the mean ± SD; **P < 0.01 compared with control using Student *t*-test.

To further determine if treatment with a combination of DDP and melatonin may be a potential therapeutic strategy to treat NPC, we examined the antitumor capability of melatonin in a xenograft nude mouse model. HONE1 cells were injected into the tail veins of nude mice, and mice were randomly assigned to four treatment groups (n = 3 per group). Treatment with DDP, melatonin, both agents, and vehicle control began2 weeks after injection, and lasted for 4 weeks (Figure 6A). While DDP or melatonin treatment alone showed some suppressive effects compared to the control group at 4 weeks (Figure 6B), combination treatment with DDP and melatonin showed a significant reduction in tumor burden (Figure 6C–6E). HE and IHC staining also showed that the combination of DDP and melatonin resulted in reduction in tumor volume, lower cell proliferation indices (Ki67-positive), and lower expression of β-catenin (β-catenin-positive) compared with DDP or melatonin alone treatments (Figure 6F). Collectively these results indicate that treatment with a combination of DDP and melatonin inhibits metastasis of NPC cells *in* *vivo*.

## DISCUSSION

**Figure 7:**
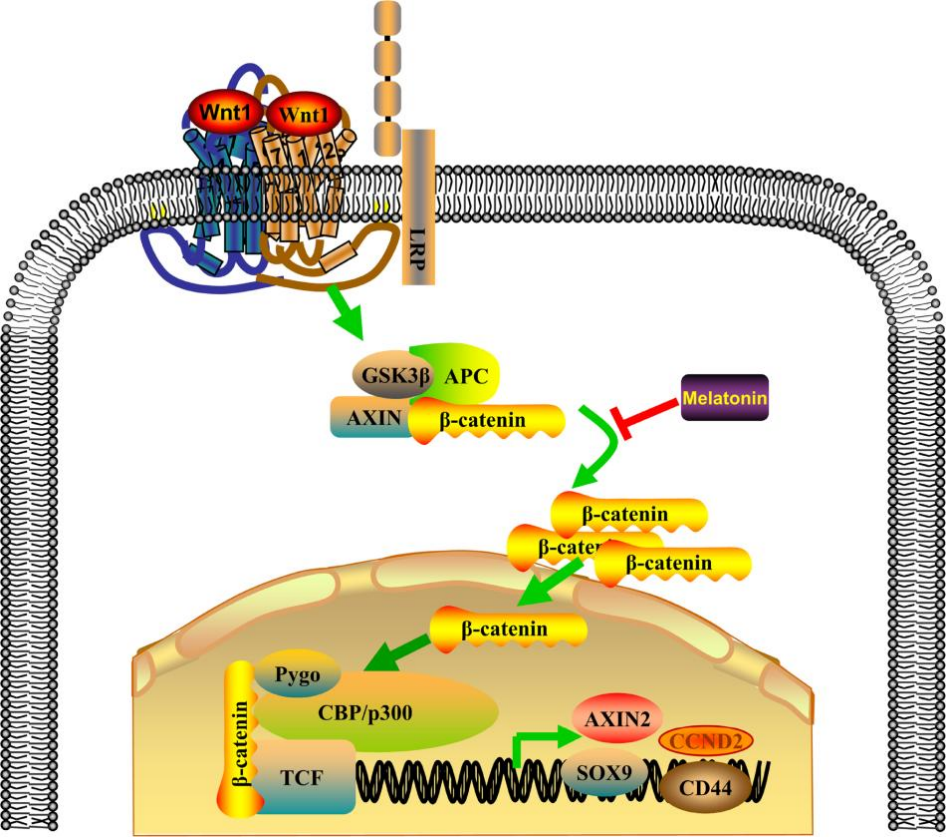
**Proposed model of the combinatorial effect of DDP and melatonin on NPC cells.**

NPC is one of the most common head and neck cancers in Southern China and Southeast Asia [24]. Most NPC cells are poorly differentiated squamous cells and are highly sensitive to chemotherapy, and chemotherapy remains the mainstay of treatment for NPC. In this study, we report that melatonin inhibited activation of the Wnt/β-catenin pathway by suppressing β-catenin nuclear translocation, reversing DDP chemoresistance of NPC cells *in vitro* and *in vivo* (Figure 7), and indicating that treatment with a combination of melatonin and DDP may be a constructive therapeutic strategy to treat NPC.

The chemotherapeutic drug DDP is a standard initial treatment for patients with LA-NPC [25–27]. Phase 2 trials have shown that gemcitabine plus cisplatin is an effective chemotherapeutic strategy in patients with NPC [28–30]. We performed a phase 3 trial which confirmed that the combination of docetaxel, cisplatin, and fluorouracil (TPF) chemotherapy to CCRT can significantly improve 3-year distant metastasis-free survival (DMFS) for LA-NPC patients [30]. However, this treatment strategy only results in a 7% improvement in control of disease metastatic. The limited effect is attributable to many factors, including extrinsic or intrinsic resistance to conventional chemotherapy.

DDP has been reported to activate the Wnt/β-catenin through some ligand-independent regulatory mechanisms, which can reduce its antitumor activity [31]. Moreover, antagonism of β-catenin reverses cisplatin resistance in DDP-treated mouse models [32]. In the current study, we showed that DDP resistance resulted in an increase in nuclear β-catenin and in the expression levels of β-catenin in NPC cells. Wnt/β-catenin activation is involved in the induction of stemness, inhibition of differentiation, and regulation of gene products that promote proliferation, migration, and invasion of NPC cells, which contributes to chemoresistance in NPC [33–35]. Therefore, the Wnt/β-catenin pathway is a potential target for developing novel therapeutic strategies for NPC.

Melatonin mitigates tumorigenesis, progression, and metastasis of many types of cancer, including breast cancer, ovarian cancer, colorectal cancer, and lung cancer [36–39]. Melatonin inhibits 12-O-tetradecanoylphorbol-3-acetate-induced cell motility by regulating expression of matrix metalloproteinase-9 (MMP-9) in NPC cells [40]. In the present study, we investigated the responses of NPC cells to treatment with a combination of melatonin and DDP. Both DDP and melatonin have been shown to individually inhibit cancer cell growth in many studies, but they have never been combined together to treat nasopharyngeal cancer. Our results showed that DDP chemoresistance in NPC cells is mediated through activation of the Wnt/β-catenin signaling pathway. We show that melatonin can reverse DDP chemoresistance in NPC cell lines and can enhance the inhibitory effects of DDP by decreasing phosphorylation of β-catenin. However, underlying mechanism for this requires further elucidation in future studies.

In conclusion, we report that chemoresistance to DDP in NPC is mediated through activation of the Wnt/β-catenin pathway, and that melatonin treatment can reverse DDP chemoresistance and enhance the antitumor effects of DDP in NPC. Therefore, combination treatment with melatonin and DDP may be a promising clinical approach to treat patients with NPC.

## MATERIALS AND METHODS

### Cell lines and cell culture

Human NPC cell lines (CNE2, SUNE1, HONE1, and 5-8F) were cultured in RPMI-1640 (Invitrogen, USA) supplemented with 5% FBS (Gibco). Cisplatin resistant cell lines, 5-8F/DDP and CNE2/DDP, were established by long-term culture in increasing concentrations of DDP, up to 2.5 μg/ml, for more than 6 months. The normal human nasopharyngeal epithelial cell line, NP69, was grown in KSF medium (Invitrogen) supplemented with bovine pituitary extract (BD Biosciences, San Jose, CA). All nasopharyngeal epithelial cell lines and NPC cell lines were generously provided by Professor Musheng Zeng (Sun Yat-sen University Cancer Center, China).

### Reagents

Cisplatin (DDP) was purchased from Selleck Chemicals (Houston, TX, USA) and dissolved in water. Melatonin was purchased from Sigma-Aldrich (St.Louis, MO, USA) and dissolved in DMSO.

### Whole transcriptome expression analysis (RNA-seq)

Total RNA was extracted from 5-8F and 5-8F/DDP cells. After removal of the RNA-later reagent, samples were stored at –80°C. RNA quality was assessed using an Agilent Bioanalyzer (Agilent Technologies, Santa Clara, CA, USA), and high-quality RNA samples (RNA integrity numbers >7.0) were used for RNA-seq analysis.

Total RNA (1 μg) was used to generate sequencing libraries using the TruSeq Stranded mRNA Sample Prep Kit (lllumina, San Diego, CA, USA) following the manufacturer’s instructions. Libraries were subjected to paired-end sequencing of 43-bp reads using a NextSeq500 System (Illumina) with a NextSeq500 High Output Kit (Illumina). The reads were aligned to the UCSC reference human genome 19 (hg19) using Spliced Transcripts Alignment to a Reference (STAR) software v2.3.1 (DNASTAR, Inc. Madison, WI, USA). Differentially expressed genes (DEGs) were detected using DESeq v1.24.0. Genes with significant expression differences in 5-8F/DDP compared with 5-8F cells were determined (|log_2_(fold change)| >1.5, adjusted *P* < 0.05).

### Gene set enrichment analysis (GSEA)

A GSEA software tool (version 2.0.13, https://www.broad institute.org/gsea/) was used to identify KEGG pathways (MSigDB, version 4.0) that are overrepresented in the genes that were upregulated or downregulated between the 5-8F and 5-8F/DDP cells. Briefly, an enrichment score was calculated for each gene set (i.e., KEGG pathway) by ranking gene expression difference.

### Western blot

Total protein was extracted from cells using RIPA lysis buffer (P0013B, Beyotime, China) or Nuclear and Cytoplasmic Protein Extraction Kit (P0027, Beyotime, China). Protein concentrations were quantified using a BCA Protein Assay Kit. Proteins were separated using SDS-PAGE, then electrophoretically transferred to membranes. Membranes were blocked with 5% non-fat milk and incubated overnight at 4°C with primary antibodies (Supplementary Table 1).

### RNA isolation and quantitative PCR

Total RNA was extracted using TRIzol regent (Life Technology, Carlsbad, USA) and converted to cDNA using a M-MLV reverse transcriptase kit (Promega). Quantitative PCR was performed and analyzed as described previously [41]. The primer sequences are shown in Supplementary Table 2.

### Immunofluorescence

Cells were fixed with methyl alcohol, permeabilized with 0.5% Triton X-100, and incubated with primary antibodies. Cells were subsequently incubated with species-matched secondary antibodies (Supplementary Table 2). Nuclei were stained with DAPI (Sigma) and fluorescence images were obtained using a confocal scanning microscope (Olympus FV1000).

### TOP/FOPFlash reporter assay

TOP/FOPFlash reporter assays were used to determine the transcriptional activity of the Wnt/β-catenin pathway. The reporter plasmids containing TOPFlash and mutated FOPFlash TCF/LEF DNA binding sites were purchased from Addgene. Briefly, NPC cells and DDP resistant cells were seeded in 96-well plates and transfected with the TOP/FOPFlash vector and pRL-TK vector (Promega). Luciferase activity was measured using the Promega Dual-Luciferase Reporter Assay System 24 h after transfection. The firefly luciferase activity in each sample was normalized to the *Renilla* luciferase activity within that sample.

### Cell viability assay

Cells (1 × 10^3^) were seeded into 96-well plates and incubated for the indicated time periods (1, 2, 3, 4, 5 days). On the indicated days, CCK-8 reagent (10 μl; Dojindo, Tokyo, Japan) was added to each well and incubated for 2 h. After incubation, the absorbance at 450 nm in each well was read using a spectrophotometer.

### Colony formation assay

Cells (0.3 × 10^3^) were seeded into 6-well plates and cultured for 1 week (CNE2 and HONE1 cells) or 2 weeks (5-8F and SUNE1 cells). Colonies that formed were fixed with methyl alcohol, stained with crystal violet, and quantified.

### Cell apoptosis

Cells were lifted from culture plastic, centrifuged, and resuspended in a PBS buffer to form a single-cell suspension. Cell death in NPC cell lines induced by melatonin was analyzed by flow cytometry using Annexin-V/propidiumiodide (PI), assays according to the manufacturer’s instructions (BD Biosciences, Bedford, USA).

### Transwell migration and invasion assays

The effects of DDP and/or melatonin on the migratory and invasive ability of NPC cell lines were evaluated by transwell assays. Cells (5 × 10^4^ or 1 × 10^5^) were suspended in 200 μL of serum-free medium and plated into transwell chambers with 8 μm pores (Corning) that were uncoated (migration assay) or coated with Matrigel (BD Biosciences) (invasion assay). Cells were cultured at 37°C for 12 hours or 24 hours. Methanol was used to fix the cells that had migrated or invaded into the lower chamber. Cells were stained with 1% crystal violet solution and manually counted.

### Tumor xenografts experiments in vivo

BALB/c nude mice (six-week old) were obtained from Charles River Laboratories (Beijing, China). HONE1-luciferase cells (1 × 10^6^) were resuspended in 200 μL serum-free medium and injected intravenously into the tail veins. After 6 weeks, the mice were randomly assigned (n = 3 per group) to receive the following treatments on a weekly schedule: Group 1 served as a control and received no treatment; group 2 received 4 mg/kg intraperitoneal DDP once a week; group 3 was treated with 25 mg/kg intraperitoneal melatonin on four consecutive days per week; group 4 was treated with 4 mg/kg, intraperitoneal DDP once a week followed by 25 mg/kg melatonin each day for four days. Treatments were continued for 4 weeks.

After euthanasia, lung tissues were fixed to determine the numbers of metastatic nodes formed on the surface of lungs. The tissues were paraffin-embedded, and serial 5-μm tissue sections were cut and stained with hematoxylin–eosin (HE) to examine metastatic nodes in the lungs as previously described [42, 43]. All animal research was conducted in accordance with the regulations approved by the Animal Care and Use Ethnic Committee of Affiliated Cancer Hospital and Institute of Guangzhou Medical University, and all efforts were made to minimize animal suffering.

### Immunohistochemistry (IHC) assay

Tissue sections were deparaffinized with xylene and rehydrated with a gradient of ethanol to distilled water. After treatment with citrate buffer (pH 6.0), the tissue sections were pre-incubated with hydrogen peroxide and blocked with goat serum (Beyotime). The sections were then incubated with primary antibodies (Supplementary Table 2) at 4°C overnight. Sections were then incubated with secondary antibody working solution (Dako, GK500705) for 1 h at 37°C. Finally, a the sections were stained with a DAB Substrate Kit (Invitrogen, USA) for 5 min to observe protein expression, as previously described [44].

### Statistical analysis

All statistical analyses were carried out using SPSS 16.0 statistical software (SPSS Inc.). All data presented as the mean ± SD were derived from no less than three independent experiments. Differences between groups were analyzed using the Student’s t test or the χ2 test. All tests were two-tailed; P < 0.05 was considered statistically significant.

## Supplementary Material

Supplementary Figures

Supplementary Tables
