# Supplementary material for: Melatonin reverses nasopharyngeal carcinoma cisplatin chemoresistance by inhibiting the Wnt/β-catenin signaling pathway
Source: Aging (Albany NY). 2020 Mar 23;12(6):5423–38. doi: 10.18632/aging.102968 (PMC7138577; doi:10.18632/aging.102968)
Supplement: Supplementary Figures [file aging-12-102968-s002..pdf]

SUPPLEMENTARY FIGURES

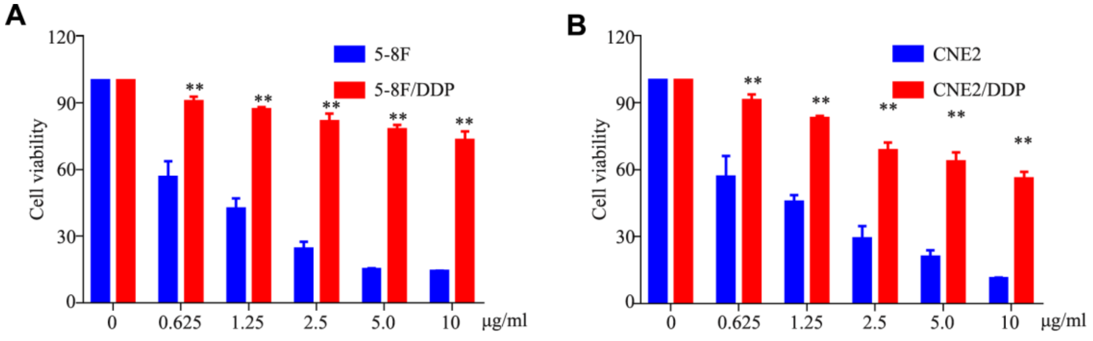

Supplementary Figure 1. Establishment of cisplatin-resistant NPC cells. (**A**, **B**) CCK8 assay was conducted in parental and 5-8F/DDP (**A**) and CNE2/DDP (**B**) cells.

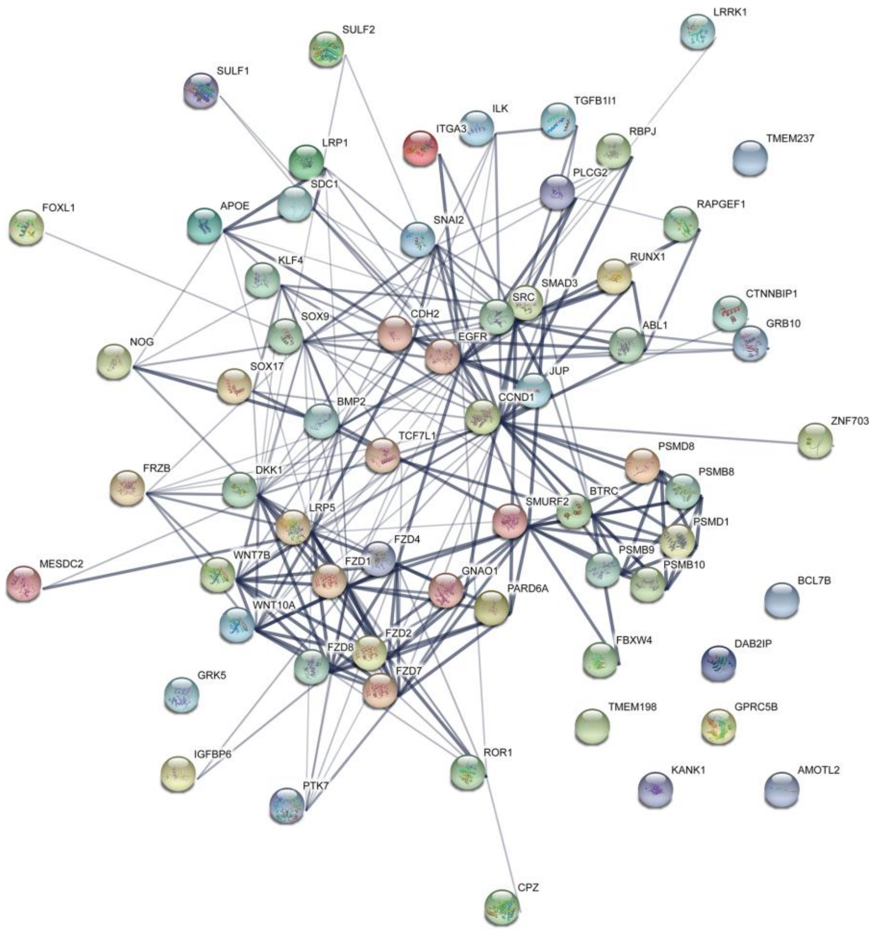

Supplementary Figure 2. Hub genes in the Wnt/β-catenin signaling pathway.
