# Supplementary material for: Melatonin reverses nasopharyngeal carcinoma cisplatin chemoresistance by inhibiting the Wnt/β-catenin signaling pathway
Source: Aging (Albany NY). 2020 Mar 23;12(6):5423–38. doi: 10.18632/aging.102968 (PMC7138577; doi:10.18632/aging.102968)
Supplement: Supplementary Tables [file aging-12-102968-s001..pdf]

SUPPLEMENTARY TABLES

Supplementary Table 1. Primers used in this study.

Gene	Sequence (5' to 3')
CCND2-F	GAGAAGCTGTCTCTGATCCGCA
CCND2-R	CTTCCAGTTGCGATCATCGACG
CD44-F	CCAGAAGGAACAGTGGTTTGGC
CD44-R	ACTGTCCTCTGGGCTTGGTGTT
SOX9-F	AGGAAGCTCGCGGACCAGTAC
SOX9-R	GGTGGTCCTTCTTGTGCTGCAC
AXIN2-F	CAAACTTTCGCCAACCGTGGTTG
AXIN2-R	GGTGCAAAGACATAGCCAGAACC
GAPDH-F	TGATGACATCAAGAAGGTGG
GAPDH-R	TTGTCATACCAGGAAATGAGC

Supplementary Table 2. Antibodies used in this study.

Antibody	Company	Catalog no.	Dilution
**Western blot**			
β-cantenin	Proteintech	21112-1-AP	1:1000
DDK1	Proteintech	51067-2-AP	1:1000
c-Myc	Proteintech	10828-1-AP	1:2000
CyclinD1	Proteintech	60186-1-Ig	1:5000
GAPDH	Proteintech	51064-2-AP	1:5000
Mouse	CST	7076	1:5000
Rabbit	CST	7074	1:5000
**Immunohistochemistry**			
Ki67	CST	9949	1:400
β-cantenin	Proteintech	51067-2-AP	1:200
**Immunofluorescence**			
β-cantenin	Life	51067-2-AP	1:2000
CoraLite594 – conjugated Goat Anti-Rabbit IgG(H+L)	Proteintech	SA00013-4	1:200
